# miR-205: A Potential Biomedicine for Cancer Therapy

**DOI:** 10.3390/cells9091957

**Published:** 2020-08-25

**Authors:** Neeraj Chauhan, Anupam Dhasmana, Meena Jaggi, Subhash C. Chauhan, Murali M. Yallapu

**Affiliations:** 1 Department of Immunology and Microbiology, School of Medicine, University of Texas Rio Grande Valley, McAllen, TX 78504, USA; neeraj.chauhan@utrgv.edu (N.C.); anupam.dhasmana@utrgv.edu (A.D.); meena.jaggi@utrgv.edu (M.J.); subhash.chauhan@utrgv.edu (S.C.C.); 2 South Texas Center of Excellence in Cancer Research, School of Medicine, University of Texas Rio Grande Valley, McAllen, TX 78504, USA

**Keywords:** miRNAs, miR-205, cancer, tumor suppressor, nanoformulation, targeted delivery

## Abstract

microRNAs (miRNAs) are a class of small non-coding RNAs that regulate the expression of their target mRNAs post transcriptionally. miRNAs are known to regulate not just a gene but the whole gene network (signaling pathways). Accumulating evidence(s) suggests that miRNAs can work either as oncogenes or tumor suppressors, but some miRNAs have a dual nature since they can act as both. miRNA 205 (miR-205) is one such highly conserved miRNA that can act as both, oncomiRNA and tumor suppressor. However, most reports confirm its emerging role as a tumor suppressor in many cancers. This review focuses on the downregulated expression of miR-205 and discusses its dysregulation in breast, prostate, skin, liver, gliomas, pancreatic, colorectal and renal cancers. This review also confers its role in tumor initiation, progression, cell proliferation, epithelial to mesenchymal transition, and tumor metastasis. Restoration of miR-205 makes cells more sensitive to drug treatments and mitigates drug resistance. Additionally, the importance of miR-205 in chemosensitization and its utilization as potential biomedicine and nanotherapy is described. Together, this review research article sheds a light on its application as a diagnostic and therapeutic marker, and as a biomedicine in cancer.

## 1. Introduction

microRNAs (miRNAs) are endogenous small (18–23 nucleotides bases) non-coding single RNAs (ncRNAs) which bind to the three prime untranslated regions (3′ UTRs) of the target messenger RNAs (mRNAs) and control the gene expression at the post-transcriptional level [1]. miRNAs are generated by Drosha and Dicer, two RNase III proteins. Biogenesis of miRNA(s) involves various steps including transcription by polymerase II, processing by Drosha in the nucleus, processing by Dicer in the cytoplasm, modification (by RNA editing, methylation, uridylation, adenylation), loading into Argonaute, which is the main protein of RNA-induced silencing complex (RISC), and targeted RNA degradation [2]. Through these processes, miRNAs can regulate various cellular functions such as cell growth, differentiation/development, and apoptosis. Dysregulation of miRNAs plays a key role in many pathological processes of various diseases including cardiovascular diseases, diabetes, neurodevelopmental diseases, inflammatory diseases, liver diseases, autoimmune diseases, skin diseases, and cancer [3]. Thus, miRNAs have received significant attention as new prognostic and therapeutic tools for the management of many diseases.

To date, a total of 2656 mature miRNAs have been identified [4]. *Lin-4* was the first miRNA to be identified three decades ago in *Caenorhabditis elegans* [5]. Later another miRNA was also identified in the same worm named as *let-7*. Both of these miRNAs were reported to negatively regulate the post-embryonic developmental processes [6]. It has been established that a single miRNA can regulate many genes which suggests that miRNAs are involved in multiple cellular pathways and functions [1]. Literature has demonstrated that impaired expression of miRNAs has been implicated in cancer initiation, progression (tumorigenesis), and metastasis [7,8].

Calin et al., [9] for the first time, has indicated that miRNAs function as tumor suppressors. This finding suggested a common deletion of miR-15a and miR-16-1 in ~65% of chronic lymphocytic leukemia patients. These miRNAs showed a negative regulation of Bcl-2 protein (an anti-apoptotic protein), frequently overexpressed in various types of cancers [10]. Later reports suggested that deregulation of miRNA(s) in cancer was suggestive of their potential role as oncogenes or tumor suppressors [11]. In general, tumor suppressor miRNAs are downregulated and oncogene miRNAs are highly expressed in human cancers [12,13]. For instance, tumor suppressive miR-145 is often downregulated in many cancers such as breast, cervical, pancreatic, and colon [14], and oncogenic miR-21 is upregulated in various cancers [15]. The fundamental function of miRNAs depends on their expression status (upregulated or downregulated) in a specific cancer type. The oncogenes can be suppressed by tumor suppressive miRNAs and the tumor suppressor genes can often be downregulated by oncogenic miRNAs. miRNAs have been documented to regulate multiple cellular processes in cancer such as cell proliferation, tumor growth, cancer invasion, and motility [16].

Cancer metastasis is the major concern in cancer treatments. Systemic metastasis accounts for up to 90% of cancer-related deaths [17]. Thus, identification of miRNAs tackling this condition is a highly unmet clinical need. To illustrate the role of some miRNAs in cancers, miR-17 to 92 cluster (miR-17, miR-18a, miR-19a, miR-19b-1, miR-20a, and miR-92a) was reported to be highly overexpressed in lung cancer and lymphoma [18,19]. This cluster targets c-Myc oncogene, controls cellular death, and proliferation via p53 tumor suppressive signaling pathway [20,21]. Additionally, in breast cancer, the family of miR-200 (miR-200a, miR-200b, miR-200c, miR-141, and miR-429) and miR-146 were found to be notably suppressed [22]. Furthermore, another cluster of miR-371-3 was identified acting as oncogenes in human cancers. For instance, in testicular germ cell tumors, miR-372-3 cluster promoted both cell growth and tumor progression by directly inhibiting large tumor suppressor kinase 2 and reversing/nullifying p53 mediated inhibition of cyclin-dependent kinase [23,24]. This miRNA cluster was demonstrated to have an oncogenic role that was derived from eleven scrutinized studies (870 participants’ sample data).

Besides having a specific role in cellular functions, many miRNAs have cancer specific expression status. One such miRNA is miR-205 which has different expression status in different cancers [25]. For example, it acts as a tumor suppressor in breast cancer [26] but functions as an oncogenic miRNA in cervical cancer [27]. Among many miRNAs, miR-205 is consistently downregulated in breast, prostate, liver, and skin cancers [28,29,30,31,32,33,34]. Thus, in this review article, we focus on the tumor suppressive role of miR-205 in different cancers, discuss its therapeutic advantages, and delineate its enhanced delivery options using nanotechnology.

## 2. miR-205 in Cancers: Friend or Enemy?

miR-205 is a highly conserved miRNA and has been identified in various species [35]. miR-205 was first identified in mouse and Fugu rubripes sequences and later it was found to be expressed in zebrafish and human [36,37,38]. The first time human miR-205 was identified by a computational study based on its conservation in mouse species [36]. miR-205 is expressed primarily in the myoepithelial cells [30]. The expression status of miR-205 is highly dependent on the cancer and tissue type, therefore, it can act either as a friend or as a foe in different cancers [39] (Table 1).

**Figure 1:**
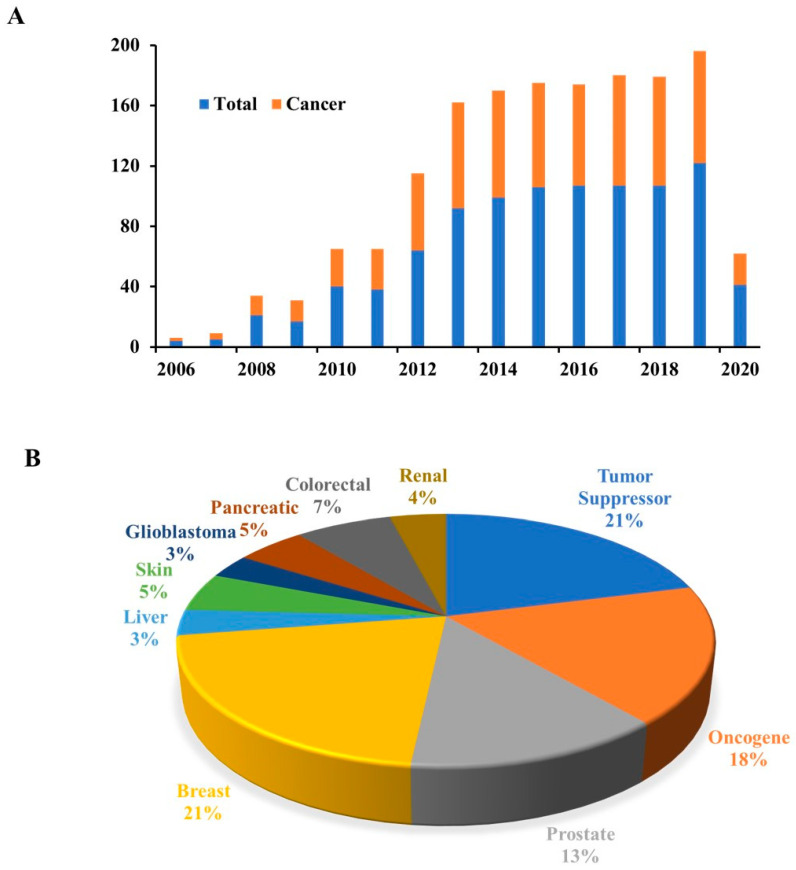
(**A**) Number of peer reviewed articles. Extracted from PubMed, and searched as miR-205, miR-205 in cancer. (**B**) Pie Chart showing distribution of miR-205 articles with different search criteria. Articles were searched for miR-205 as tumor suppressor in cancer, miR-205 as oncogene in cancer and miR-205 in different cancers.

In 2006, research studies were initiated on miR-205 but significant attention was only received after 2010 (Figure 1A). Several miRNA profiling studies (Figure 1B) suggested that miR-205 has cancer specific expression status. It is suppressed in prostate, breast, skin and liver cancers, whereas in lung, ovarian, head and neck and bladder cancers it is upregulated, however, there is no significant change in its expression in colon cancer. Additionally, the expression of miR-205 is very low in the normal epithelial cells and overexpressed in squamous cell carcinomas and adenocarcinomas [67,68]. Teta et al., [69] published that the expression of miR-205 is very epithelial specific as it was found to be overly expressed in skin epidermis but not in hair follicle. Moreover, overexpression of miR-205 plays a crucial role in the stem cells differentiation of mammary gland [70,71].

In non-small cell lung cancer, miR-205 induces carcinogenesis by promoting Protein Kinase B signaling via targeting PH domain Leucin rich repeat Protein Phosphatase 2 (PHLPP2) [61]. Other studies have also revealed that in some cancers, miR-205 regulates tumor initiation and development/progression, and also develops resistance in cells for chemotherapies [72,73]. Supporting the oncogenic role of miR-205, Xie et al., [27] have suggested that miR-205 is highly upregulated in cervical cancer samples along with downregulated expression of two of its downstream targets, CYR61 and CTGF, these two markers play an essential role in cell proliferation and migration. Furthermore, miR-205 was documented as overexpressed in squamous cell carcinoma which promoted AKT signaling by targeting Src Homology domain 2 containing Inositol 5 Phosphatases 2 (SHIP2) [74]. miR-205 was identified to induce cell proliferation in endometrial cancer by suppressing PTEN, and overexpression of this miRNA was correlated with the poor survival of the patients, thus, acting as an oncogene [51]. Nasopharyngeal Carcinoma (NPC) is highly radioresistant and this phenomenon was further found to be promoted by the overexpression of miR-205 in this cancer. miR-205 was also linked with tumor initiation in NPC [75].

On the other hand, many reports suggested the tumor suppressive role of miR-205 as ectopic expression of miR-205 diminished the Androgen Receptor (AR) and the Mitogen-Activated Protein Kinase (MAPK) signaling pathways, and further inhibited the tumor development in prostate cancer [76]. Majid et al., [65] also elaborated miR-205 as a tumor suppressor in renal cancer as overexpression of this miRNA reduced the expression of Src Family of protein Kinases (SFK) and its associated oncogenic signaling. Overexpression (Ectopic) of miR-205 in breast cancer cells explained the anti-growth properties of this miRNA as noticed by reduced proliferation and clonogenicity via inhibited ERBB3 which was found to be upregulated in these cells [77]. According to another report [62], miR-205 decreased cellular proliferation in melanoma by regulating AKT phosphorylation through E2F1 inhibition.

**Figure 2:**
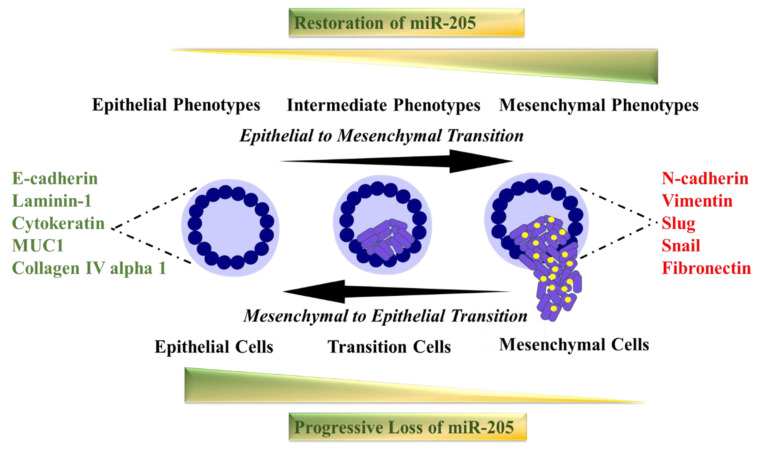
Role of miR-205 in EMT. Loss of miR-205 is a common phenomenon during the Epithelial to Mesenchymal Transition (EMT). Reversal of EMT is possible once miR-205 is restored in these cells via supplementing it through nanotechnology.

miR-205 has evolved as a major regulator of Epithelial to Mesenchymal Transition (EMT) (Figure 2). EMT is a crucial and essential feature of cancer progression and metastasis [28,78,79]. EMT has been well characterized by decreased E-cadherin and upregulated N-cadherin, and the expression levels of miR-205 were found to be repressed in cancer cells during the EMT process [42]. On the other hand, ectopic expression of miR-205 reversed the EMT process by modulating the expression of its markers (increased E-cadherin and inhibited N-cadherin) [28,42]. E-cadherin is required to maintain the epithelial characteristics of cells and is often inhibited by Zinc finger E-box Binding homeobox 2 (ZEB2) which is a direct target for miR-205 [42,79].

Lai et al., [80] proposed miRNA-mediated gene regulatory networks through mathematical modelling for various miRNA-mediated feedback loops and feedforward loops. Although it discusses numerous aspects, the EMT regulatory network with respect to miRNAs is highly relevant in our review article context. This simulation model showed that the epithelial phenotype corresponds to high levels of the miRNAs (miR-34 and miR-200) and low levels of the transcriptional factors (SNAIL and ZEB) whereas the mesenchymal phenotype corresponds to high levels of the transcriptional factors and low levels of the miRNAs. In addition, this approach provides insights into various aspects including (i) implementation of miRNA cooperativity via targeting shared pathway(s) or common/shared genes (for potential therapy), (ii) evaluation of miRNA cooperativity in cancer therapeutics either as a single miRNA/combination of miRNAs or in combination with anti-cancer agents, (iii) possibility to extent the miRNA cooperativity through the biological data feed from multiple sources to predict cooperative miRNAs regulating cancer genes. Moreover, this team also implemented experimentally verified cooperative miRNA via targeting of a shared pathway or of a shared protein-coding gene [81]. Furthermore, this study delineated a systems biology approach to investigate the application of miRNA cooperativity in cancer therapy.

In addition to modulating EMT pathway, it was reported that miR-205 attuned cellular invasion and migration by targeting and inhibiting many other markers such as Lipoprotein Receptor-related Protein 1 (LRP1) which enhanced the expression of Matrix Metalloproteinase 2 and 9 (MMP2 and MMP9), resulting in increased migration and invasion in glioblastoma and lung cancer [82,83]. Moreover, miR-205 controls invasion in breast cancer by inhibiting Vascular Endothelial Growth Factor A (VEGFA), a known angiogenesis marker [77]. These accumulating evidence(s) supports the hypothesis of the dual role of miR-205, able to act as an oncogene or a tumor suppressor depending on the cellular context. In this review, we discuss the tumor suppressive role of miR-205 in detail in different cancers.

## 3. Tumor Suppressive Role of miR-205 in Different Cancers

### 3.1. Prostate Cancer

Prostate cancer stands as the second most leading cause of cancer associated deaths in men in the United States [84,85]. Many reports have confirmed that miR-205 is suppressed in prostate cancer and acts as anti-tumor marker [58,76,86]. A comparative study done on metastasis and non-metastasis patients’ tissue samples with Real-time quantitative PCR (qRT-PCR), explained that miR-205 was downregulated more in metastatic samples than the non-metastatic counterparts. Interestingly, in this study, levels of miR-205 were also found to be remarkably downregulated in castration resistant patients’ samples compared to androgen naïve patients’ samples. Further, histopathological analyses by utilizing in situ hybridization for patients derived prostatic tissue samples revealed that miR-205 was distinctly expressed in epithelial cells but not in stromal cells. Cellular location analysis of miR-205 also resulted in a similar finding as the miRNA was found to be located in the cytoplasmic region of epithelial cells [58]. In correlation with these reports, a miRNA profile experiment demonstrated that miR-205 was comparatively suppressed in prostate cancer samples than the normal control samples, and this downregulation of miR-205 was cancer stage dependent [87]. Prostate cancer/tumor stage and Prostate Specific Antigen (PSA) expression levels have also been correlated with miR-205 status [88]. miR-205 was mostly overexpressed in tissue samples with Benign Prostatic Hyperplasia (BPH) and the expression reduced as the grade of the disease progressed, indicating that the expression of miR-205 was inversely correlated with the presence of PSA [58].

PSA was reported to be regulated by Androgen Receptor (AR) signaling [89] and AR transcript levels were found to be reduced after the transient expression of miR-205 in androgen dependent VCaP and 22Rv1 prostate cancer cell lines [58]. Androgen receptor signaling was reported as one of the significant signaling pathways that contributes to prostate cancer development [58,90]. Moreover, it was confirmed by a luciferase reporter assay that miR-205 directly binds to the 3′ UTR of AR and the ectopic expression of miR-205 decreased the expression of the receptor at mRNA levels but not much at protein levels in LNCaP prostate cancer cell line [58]. One possible reason for this phenomenon was that some other signaling pathways such as PI3K-AKT pathway may interfere with AR protein and degrade its phosphorylation [91]. Once the initial tumor regression is performed with the available AR deprivation treatment, prostate cancer progresses to the castration resistant form, called CRPC [58,90]. Overexpression of miR-205 in a nude mouse xenograft model reduced the tumor progression and cellular proliferation as shown by reduced Ki-67 expression on the excised tumor tissue sections [87]. Bcl-2 which is an anti-apoptotic protein, plays a pivotal role in prostate cancer and has been linked with poor prognosis and relapse of the disease [92,93,94,95]. This anti-apoptotic gene was found to be a significant target of miR-205. Moreover, overexpression of miR-205 induced apoptosis and decreased mitochondrial membrane potential and cell growth via targeting/reducing Bcl-2 expression in prostate cancer cells [86].

According to The Cancer Genome Atlas (TCGA) database, High Mobility Group Box 3 (HMGB3) is a gene which is responsible for shorter prostate cancer free survival and involved in prostate cancer pathogenesis. HMGB3 was found to be downregulated in prostate cancer cells when these cells were transfected with miR-205 as demonstrated by a luciferase reporter assay [96]. Moreover, upregulation of miR-205 has direct inhibitory effects on Centromere Protein F (CENPF) which acts as one of the major oncogenes in prostate cancer and leads to cancer aggressiveness [97].

miR-205 plays an important role in the regulation of EMT [42]. c-SRC belongs to a family of non-receptor tyrosine kinases called SRC Family Kinases (SFKs) and is by far the most studied protein of its family. SRC family proteins play a major role in multiple signaling pathways (RAS/RAF/ERK1/2, PI3K/AKT/HIF-1α, FAK/p130CAS/MMP9, β-Catenin/c-Myc/Cyclin D1, STAT3/c-Myc/Cyclin D1, and RAC/NADPH) which are associated to tumorigenesis, cell proliferation, migration, invasion, and motility/metastasis [98]. This study demonstrated that miR-205 can efficiently inhibit the constitutive activity of p-FAK, p-ERK1/2, c-Myc, and Cyclin D1 in cancer cells with suppressed p-c-SRC expression.

A luciferase reporter assay revealed that restoration of miR-205 significantly reduced the expression of c-SRC. Furthermore, transient overexpression of miR-205 also inhibited the expression of FAK/ERK1/2 pathway, which is crucial for tumor development, via targeting/reducing c-SRC [87]. Tucci et al., illustrated that miR-205 targeted ZEB1 protein which was upregulated in prostate cancer and facilitated tumor metastasis via EMT. In the presence of miR-205, p63 inhibited ZEB1 and E-cadherin, and elevated N-cadherin. Reinstatement of p63 in prostate cancer cells also resulted in an elevation of miR-205 expression [99]. Similar to miR-205, p63 is also a tumor suppressor protein, a homolog of p53 [100], and not expressed in prostate cancer cells [99].

miR-205 is also associated with autophagic events in prostate cancer [101]. Tumor Protein p53 Inducible Nuclear Protein 1 (TP53INP1) was reported to enhance the autophagy by directly targeting its two main regulators LC3 and ATG8 family proteins [102] as TP53INP1 was upregulated in prostate cancer [103]. Later, transiently overexpressed miR-205 in prostate cancer cell lines determined that miR-205 decreased the expression of TP53INP1 post transcriptionally (protein expression) but did not show any effects on mRNA/gene levels. Furthermore, upregulation of miR-205 enhanced the radiosensitivity and suppressed the irradiation induced autophagy in prostate cancer cells by inhibiting the lipidation of LC3-III and restoring the expression of p62 [104]. There are other reports as well that established miR-205 as a sensitizer of irradiation by targeting various molecules such as PTEN [105], ZEB1, and Ubc13 [106].

### 3.2. Breast Cancer

Breast cancer is the second most common cancer of women [107]. Mammogram and ultrasound are the standard screening modalities for the detection of this disease [108]. Early detection of breast cancer lesions is the main goal of clinicians to successfully manage the cancer and to reduce the mortality rate. Over the past years, researchers have found several biomarkers that aid in early detection of breast cancer. Moreover, miRNAs that are tumor specific and serve as novel biomarkers have become a newer tool for the identification of cancer.

A study done on serum samples of 58 breast cancer patients (stage I and II) and 93 healthy controls utilizing qRT-PCR revealed that the expression of miR-205 was higher in healthy control samples than in breast cancer counterparts, and there was no significant difference found in miR-205 expression between stage I and II [109]. Neoadjuvant Chemotherapy (NAC) which is given before the main surgery, plays a critical role in the successful surgical removal of breast tumors as it permits the effects of chemotherapeutic drugs on the tumor to be visualized [110,111] and allows the physicians to make better assessment of the possible future therapies. TAC [Taxotere^®^ (docetaxel), Adriamycin^®^ (doxorubicin), Cytoxan^®^ (cyclophosphamide)] is a widely accepted and used neoadjuvant chemotherapy for breast cancer [112].

A study with 30 human breast cancer tissue/biopsy samples, showed a positive correlation between the presence of miR-205 and NAC response rate, suggesting that higher expression of miR-205 was associated with the enhanced sensitivity of TAC therapy in breast cancer samples [43]. Additional studies also reported that miR-205 was downregulated in 60 human breast cancer tissue samples compared to the normal counterparts [113] as well as in HER2 negative cells [114].

Moreover, ectopic expression of miR-205 inhibited cellular proliferation and arrested cell cycle at G0-G1 phase in drug resistant breast cancer cells with and without the abovementioned drugs (inhibition of these cellular functions was more profound when drugs were used with overexpressed miR-205) [43]. A bioinformatic analysis identified that the prospective targets for miR-205 could be VEGFA and Fibroblast Growth Factor 2 (FGF2) [43,77]. VEGFA and FGF2 are two oncogenic growth factors overexpressed in many cancers including breast cancer [115,116]. miR-205 directly binds to the 3′ UTR site of these growth factors and reduces their expression in drug resistant breast cancer cells [43].

HER3 (Human Epidermal Growth Factor Receptor 3) was also reported as one of the direct targets for miR-205, determined by TargetScan and miRbase [113]. HER3 promoted cellular proliferation and motility/invasion in breast cancer [117,118]. miR-205 directly binds to HER3 at 3′ UTR and overexpression of miR-205 suppressed the HER3 protein expression levels along with modulating the expression of two other key players of cellular apoptosis, resulting in reduced expression of Bcl-2 and restored expression of Bax (pro-apoptotic). This study suggested that upregulation of miR-205 negatively impacted (inhibited) the proliferation and migration of breast cancer cells as determined by cell proliferation and wound healing scratch assays, respectively [113].

Many miRNA target prediction databases, mainly TargetScan (TargetScanHuman, prediction of miRNA target, http://www.targetscan.org/vert_72/) [119,120], miRDB-miRNA Target Prediction Database (mirdb.org) [121], miRBase (MANCHESTER 1824, www.mirbase.org) [122,123,124] and others are heavily utilized by research groups to determine the new potential targets/biomarkers and to direct their research in a novel direction. Based on TargetScan predictions, several other direct targets of miR-205 were also identified by different groups. Among many, angiomotin (AMOT) and Endoplasmic Reticulum Protein 29 (ERp29) are the direct targets for miR-205 in breast cancer and miR-205 binds to the 3′ UTR site of both genes [125,126]. Moreover, overexpression of miR-205 was found to be inversely correlated with AMOT and ERp29, resulting in the downregulation of both molecules [125,126]. AMOT has previously been reported as an oncogene in breast cancer [127] which promotes migration and angiogenesis [128]. Interestingly, transiently overexpressed AMOT was indicated to notably impair the inhibitory effects of miR-205 on the cellular proliferation and invasion of breast cancer cells [126].

miR-205 has been widely studied for its role in the regulation of EMT in many cancers [42]. miR-205 directly targets ZEB1, ERBB3, and SIP1 proteins, and modulates breast cancer invasion and migration. The overexpression of miR-205 disrupted the expression of E-cadherin and Protein Kinase C Epsilon along with the other regulators of EMT, hence, resulting in inhibited migration and invasion [28]. A study by Chao et al., [129] confirmed an epigenetic regulatory role of miR-205 by repressing the ligand jagged 1. Ligand jagged 1 secretes from the tumor microenvironment and promotes cancer-associated stem cell phenotype of tumor cells. This study demonstrated that knockdown of miR-205 in mammary epithelial cells, not only disrupted the cell polarity and self-renewable capacity but also promoted the EMT (ZEB1, NOTCH2) features and enhanced the stem cell-like (CD24^−^ and CD44^+^) population. In addition, this study demonstrated that mice spontaneously developed mammary lesions when miR-205 was deficient, whereas its activation led to abrogate the stemness in these mammary glands. Similarly, another study suggests that miR-205 can efficiently diminish the stem cell self-renewal capability by regulating YAP and Wnt signaling [130]. Collectively, these findings suggest a tumor suppressive role of miR-205 in breast cancer and that, it is a potential biomarker and therapeutic target.

### 3.3. Liver Cancer

Liver cancer is one of the deadliest diseases and ranks as fifth most common cancer in men and seventh in women [131,132]. Five-year survival rate for all stages of liver cancer is also very poor with only about 18% [132]. The current available treatment regimens for liver cancer are liver transplant, surgery, chemo and/or radio therapy, ablation therapy, and embolization therapy [133]. Pathogenesis of liver cancer involves various steps and many risk factors such as chronic hepatitis, type 2 diabetes, alcohol abuse, non-alcoholic fatty liver disease, and aflatoxin [134,135]. Currently, there are not very many drug treatment options available for liver cancer except one molecular targeted drug known as kinase inhibitor sorafenib which is highly associated with drug resistance, more than 50% [136].

Zhang et al., [137] proved the tumor suppressive role of miR-205 in liver cancer (Hepatocellular Carcinoma, HCC), as miR-205 was significantly downregulated in liver cancer tissues. They also reported low expression of miR-205 in the Hepatitis B Virus X protein (HBx) induced liver cancer mouse model. The group demonstrated that in 33 human liver cancer samples, with Hepatitis B Virus (HBV) correlation, expression of miR-205 was remarkably downregulated compared to normal liver samples. In the agreement with a previously published report [138], it was further validated that HBx was a direct target for miR-205, identified by a miRNA target prediction software, and correlated inversely with this miRNA. Additionally, hepatitis B virus protein was shown to decrease the expression levels of miR-205 in liver cancer cells [137]. Another study performed on human liver cancer tissues with qRT-PCR revealed that levels of miR-205 were decreased in the serum samples of liver cancer compared to cirrhosis and normal counterparts. Furthermore, miR-205 showed improved detection specificity and sensitivity compared to the Alpha fetoprotein (AFP) in liver cancer patients versus cirrhosis versus normal controls. AFP is the standard diagnostic tool for cirrhosis associated liver cancer [139].

The liver is the prime organ for metabolic activities in the body and thus, liver cancer is often linked with metabolism reprogramming [140]. In addition, the liver carries out many significant tasks as metabolism/breakdown, synthesis, storage, and redistribution of proteins, lipids, and carbohydrates [141]. Acyl-CoA Synthetase Long chain family member 1 (ACSL1) is a critically important gene for lipid metabolism in the liver and is also a target gene for miR-205 as accessed by TargetScan and microRNA.org [47]. Additionally, in vitro investigations revealed that ectopic overexpression of miR-205 led to the inhibition of ACSL1 both transcriptionally and translationally. Contrarily, suppressed levels of miR-205 elevated the expression of ACSL1 [47]. Moreover, the role of miR-205 in lipogenesis is dependent on ACSL1 expression in liver cells as enhanced levels of ACSL1 (due to low miR-205) increased the triglyceride levels [37]. One report even suggested that liver cancer leads to an imbalance in metabolism of lipids and lipoproteins [142].

### 3.4. Skin Cancer

Skin cancer, mainly comprised of a degree of progression in the severity of the disease, primarily includes basal cell carcinoma, squamous cell carcinoma, and melanoma [143,144]. Melanoma is the deadliest form of the commonly known skin cancers with a significantly increasing incident rate, globally [145]. Similar to many other cancers, miRNAs serve as viable biomarkers in the diagnosis and prognosis of melanoma [146]. A lesser deadly and non-melanoma form of skin cancer, squamous cell carcinoma is often termed as cutaneous Squamous Cell Carcinoma (cSCC) and is the second most common skin cancer after basal cell carcinoma [147,148].

To determine the expression of different miRNAs that are associated with skin cancer grade progression and aggressiveness, a comparative study was done on cSCC and malignant skin cancer cell lines. The group identified miR-205 as the most differentially expressed miRNA in these cell lines with a predominate expression pattern in the basal and suprabasal layers of the skin which is in accordance with other previously published reports [149]. miR-205 was constantly found to be expressed more in undifferentiated areas and in tumors with aggressive growth and invasive pattern. Further, as p63 is a well-known grading marker for the epithelial differentiation, the group found that p63 was expressed frequently in poorly differentiated tumors and more commonly in undifferentiated areas. More importantly, these tumors also exhibited miR-205 expression. A logistic regression model revealed that the expression of miR-205 was related to poor clinical outcome such as recurrence of local cSCC [150]. This study presents no expression of miR-205 in patients’ samples. Histopathological investigation [79,151] confirmed that miR-205 was significantly expressed more in invasive cSCC than in situ cSCC, therefore, it is a potential prognostic marker for skin cancer [152]. Moreover, a huge comparative microarray study identified 470 well annotated human miRNAs and 265 additional miRNAs. In this analysis, miR-205 was found to be significantly downregulated in melanoma samples as compared to benign naevi samples, as well as in A375 malignant melanoma cell line [153]. Similar to this report, another microarray study confirmed that miR-205 was specifically suppressed in metastases samples of melanoma and the expression decreased from nevus to primary to metastatic melanomas. These microarray findings were also consistent with further qRT-PCR analysis as nevi samples had significantly more expression of miR-205 than the primary and metastatic samples, indicating that it acts as a diagnostic marker for skin tumor/cancer progression [62].

Using the miRNA prediction database, two possible direct targets of miR-205 were identified as E2F1 and E2F5, and miR-205 was shown to have a direct binding site at 3′ UTR of both genes. Moreover, E2F1 and E2F5 were inversely correlated with miR-205 in melanoma cell lines as the expression of these two proteins was elevated in melanoma cell lines while miR-205 was found to be suppressed. Furthermore, when miR-205 was transiently overexpressed in these cells, levels of E2F1 and E2F5 were significantly decreased [62]. E2F1 is one of the most important and best characterized members of the E2F family in melanoma [154] and known to activate AKT cell survival pathway [155]. Overexpression of miR-205 reduced the expression of AKT at ser-473 in the cell lines which had decreased levels of E2F1. Further, miR-205 inhibited the phosphorylation of BAD and Caspase 9 [62] as both were reported to be activated by AKT [156]. E2F1 also plays an important role in cell cycle regulation [157]. Stable expression of miR-205 in C8161.9 melanoma cell line, resulted in cell cycle arrest at the G2M phase with an increased population of cells at the sub G1 apoptotic phase. Interestingly, some cell senescence characteristics were also shown by these miR-205 overexpressing cells. Further evaluations revealed that these cells showed significantly high staining/presence of SA-β-Gal (Senescence-Associated β-Galactosidase) which is a standard marker of senescence [62].

Another study reported that overexpression of miR-205 suppressed the cellular motility (migration and invasion) and proliferation both in vitro and in vivo. Additionally, cells overexpressing miR-205 exhibited more epithelial like morphological characteristics [63]. miR-205 has been extensively studied and reported as a mainstream regulator of EMT [42,79]. E-cadherin has lower levels in EMT and its expression is regulated by ZEB1 and ZEB2, two known direct targets of miR-205 [158]. miR-205 overexpression elevated the E-cadherin levels and lowered the expression of ZEB2, resulting in maintained cellular migration and invasion [63]. It is of importance to note that decreased expression of E-cadherin during EMT contributes to increased cellular migration and invasion in melanoma [159,160] and this entire process is hindered by the overexpression of miR-205 [63]. Another report also suggested that overexpression of miR-205 in many melanoma cell lines including WM35, WM793, WM115A, and 1205Lu decreased the invasion in these cells [79]. These studies, all together, suggested that as skin deregulation (navi) progresses towards the higher grade of cancer such as melanoma, expression of miR-205 becomes downregulated which argues for its tumor suppressive role in melanoma/skin cancer.

### 3.5. Glioblastoma

As per the World Health Organization (WHO) cancer grading system, Glioblastoma is a high grade or IV grade glioma (tumor of brain) and associated with a high number of incidences and deaths as it is responsible for around 47% of the total Central Nervous System (CNS) related malignancies. The relapse time for glioblastoma patients is very poor and short as most patients relapse within 7 months from the time of first diagnosis and the 5-year survival rate also remains only about 4–5% [161,162]. Surgery along with chemo and radio therapies remains the primary tool for treating glioblastoma [163]. Many research groups have reported that there are several miRNAs that play a critical role in the pathogenesis of glioblastoma [164,165].

miR-205 was identified as a tumor suppressor miRNA in glioma and was correlated with disease initiation and progression [50,166]. miR-205 was significantly upregulated in normal brain samples and showed a remarkable degree of suppression as glioma tumors progressed from grade I to grade II to grade III. Further, these results were consistent when a similar comparative study was performed with different glioma cell lines, miR-205 was reported to be downregulated in these cancerous cells [50]. Furthermore, the serum samples of glioma patients and their normal healthy counterparts (12 samples in each group), at the screening phase, revealed the notably reduced expression of miR-205 in glioma serum samples compared to normal controls. Additionally, a similar comparison was done on a larger number of samples, at the validation phase of the study, also suggesting the same findings as the expression of miR-205 in serum samples of cancer patients was significantly downregulated. Moreover, in correlation with the data from glioma tumor cells/tissues, expression of serum miR-205 was also cancer grade dependent. Normal healthy controls had a higher expression of serum miR-205 than glioma samples. In addition, low-grade cancer samples showed more serum miR-205 than the high-grade samples. Further, downregulation of miR-205 in glioma was also found to coincide with poor overall survival of the glioma patients as measured by a Kaplan Meier analysis [166].

Ectopic expression of miR-205 achieved by using a mimic for miR-205, inhibited the cellular proliferation and colony forming ability of glioma cell lines when compared to normal and scramble controls. Further, overexpression of miR-205 was found to block the cell cycle progression of these cells at G0/G1 phase and the number of Annexin V/PI (double stain for apoptotic dead cells) stained cells also increased, suggesting that miR-205 restoration in glioma cells not only hindered the ability of these cells to progress through cell division/multiplication but also initiated the apoptotic cell death. Moreover, this transiently overexpressed miR-205 was also reported to decrease the invasiveness of these glioma cells, indicating less metastasis of the cancer [50].

VEGFA is a protein that induces the formation of new blood vessels and is often upregulated in the majority of cancers. Available literature provides the evidences that inhibition of VEGFA resulted in reduced cellular invasion and metastasis [167]. Glioma cancers have also reported the overexpression of VEGFA protein which is tumor grade based and miR-205 directly binds to the 3′ UTR site of VEGFA gene, and further, downregulates the expression of VEGFA at both gene and protein levels [50].

Yes Associated Protein 1 (YAP1) is a transcriptional regulator that plays a crucial role in regulating cellular proliferation and migration in cancer [168] and was identified as another direct target for miR-205 through bioinformatics analyses utilizing TargetScan and miRanda prediction tools [49]. A luciferase reporter assay demonstrated that miR-205 directly binds to the YAP1 gene at 3′ UTR and the overexpression of miR-205 significantly reduced the expression of this transcriptional factor at both gene and protein levels as confirmed by qRT-PCR and Western blotting analyses [49]. Considering the low levels of miR-205 in glioma and its association with cancer development, this miRNA may be utilized as a potent diagnostic biomarker and can also serve as a therapeutic target.

### 3.6. Pancreatic Cancer

Pancreatic cancer is a highly aggressive cancer with limited therapeutic outcome. More than 80% of Pancreatic Ductal Adenocarcinoma (PDAC) patients are diagnosed at an already regional or distant metastasized stage. In a small sample cohort, miR-205 was found to be low in the main tumor and the tumor buds compared to the normal tissues [169]. Many pancreatic cancer cell lines (AsPC-1, BxPC-3, PANC-1, SW1990, HS-766T) showed 0.25–0.5 relative expression of miR-205 with respect to normal human pancreatic duct epithelial cell line (HPDE6-C7) [170]. An earlier study identified that miR-205 was highly downregulated compared to other miRNAs (miR-215, miR-134, miR-7, and miR-32) in MIA PaCa-2 cancer stem cell population [171]. Additionally, its replenishment therapy reduced the cancer stem cell markers and induced the sensitization to gemcitabine (GEM) therapy. Another study determined that the expression of miR-205 was downregulated when BxPC-3 cells became resistant to Gemzar (GEM) treatment [172]. Mittal et al., [173] generated miR-205 micelles for clinical effectiveness of GEM in pancreatic cancer. Furthermore, the same group reported that combination treatment of GEM and miR-205 [55], and EGFR-targeted co-delivery of miR-205 and GEM [174] effectively reduced the tumor growth in orthotopic mouse models. In contrast to the tumor suppressive role of miR-205 in pancreatic cancer, there are some studies that suggest that it has oncogenic and ambiguous roles in pancreatic cancer [175,176,177]. Additionally, few more studies confirmed miR-205 as a biomarker for pancreatic cancer [178,179].

### 3.7. Colorectal Cancer

Colorectal cancer (CRC, also referred to as colon cancer, bowel cancer, or rectal cancer) is the development of cancer from the colon or rectum. CRC is the third most common cancer and the third leading cause of cancer-related deaths in the US. A differential expression of miR-205 was found in prospectively collected CRC samples and their normal adjacent tissues (*n* = 40) [180]. However, the relative expression of miR-205 in tumor samples showed a significant decrease (0.04 ± 0.07) in relation to some normal tissues (0.07 ± 0.07). miR-205 exhibited upregulation during the tumorigenesis but it is not significant. Similarly, miR-205 showed the reduced relative expression in 20 paired CRC tissue samples compared to the adjacent non-tumor tissues [66]. Further, its relative expression was downregulated in CRC cell lines (SW480, ~0.3; HT29, ~0.4; HCT116, ~0.6) in comparison with a normal colon epithelium cell line (FHC, 1.0). Additionally, this study suggested that miR-205 functions as a tumor suppressor by inhibiting proliferation, invasion, and migration due to effectively targeting cAMP responsive element binding protein 1. A study attributed the anti-proliferative role of miR-205 in CRC by the ERβ-miR-205-PROX1 mechanism [181]. Activation of Proteinase-Activated Receptor 2 (PAR2) was reported to promote cell migration in various cancers, including CRC. A recent study supported that PAR2 activation decreased miR-205 which in result increased the Bone Morphogenetic Protein Receptor type IA (BMPR1A) leading to increased cell migration [182]. Chen et al., [183] proved the potential role of miR-205 in the developmental process of CRC through Protein-Tyrosine Kinase 7 (PTK7). This study confirmed that the expression of miR-205 was lower in HT29 and SW480 CRC cell lines compared to other miRNAs (miR-409, miR-495, miR-5688, and miR-503). miR-205 was shown to have negative correlation with PTK7 in CRC tissues. Additionally, miR-205 was involved in FBXW7α (tumor associated macrophage polarization) [184], Long non-coding RNA (lncRNA) NEAT1-VEGFA [185] and, lncRNA ZEB1-AS1 and YAP1 [186] signaling axes for inhibiting proliferation, migratory and invasive characteristics, and promoting apoptosis in CRC. All these events confirm the tumor suppressive role of miR-205 in CRC.

### 3.8. Renal Cancer

Renal cell carcinoma (RCC) occurs as the seventh most common cancer in the US. It contributes about 14,830 deaths in 2020 in the US alone. The overall 5-year survival rate is about 60%. A study by Majid et al., [65] demonstrated that the relative expression of miR-205 was significantly downregulated in RCC tumor tissues (*n* = 32) compared to normal samples (*n* = 32) (*p* ≤ 0.001). This study also presented ~25-fold low expression of miR-205 in A498, ACHIN, Caki-1, and 769-P human RCC cell lines compared to a non-malignant renal cell line, HK-2. Further, miR-205 overexpression was able to induce apoptosis and cell cycle arrest, and impair cell viability, migration and invasion of RCC cell lines. Both in vitro and in vivo studies confirmed that miR-205 suppressed SRC family members (Src, Lyn, and Yes mRNA or protein) and negatively regulated Ras/Raf/ERK1/2 pathway. Another clinical study confirmed the lower miR-205 relative expression in 60 RCC patients’ tissues with respect to adjacent normal tissues (*p* < 0.01) [187]. This study further delineated the relationship between miR-205 expression and clinicopathological features of tissue samples: PT stage (T1, 3.38 ± 1.83 vs. T2–4, 3.67 ± 2.14), clinical stage (stage I, 3.98 ± 2.37 vs. stage II–IV, 3.85 ± 2.21), metastasis (no metastasis, 4.21 ± 2.56 vs. metastasis, 3.29 ± 3.32), and recurrence (no recurrence, 3.86 ± 2.09 vs. recurrence, 3.06 ± 2.52). Moreover, 80% of RCC patients who had higher miR-205 survived for 40 months compared to those who had reduced miR-205 (40% survival for 40 months). A study based on functional, biochemical, and bioinformatic approaches demonstrated that Ankyrin repeat and single KH Domain 1 (ANKHD1) induced the renal cancer cell proliferation [188]. Through KH domain, ANKHD1 physically interacts with miR-205.

## 4. Therapeutic Applications of miR-205

Considering the significance of miRNAs in the pathogenesis of many cancers, their therapeutic aspect is highly valuable. The ability of miRNAs to regulate many genes rather than just one selected target, makes miRNAs an even more attractive therapeutic tool for cancer researchers. More importantly, the manipulation of miRNAs (knockdown or overexpression) has been heavily investigated and resulted in promising approaches to manage various tumor types. The first clinical trial with a miRNA was introduced for treating hepatitis C virus infection using a Locked Nucleic Acid (LNA) modified anti-miR-122 in Denmark by Santaris Pharma (miravirsen, SPC3649) [189]. Further, utilizing this LNA modified anti-miR-122 (miravirsen, ClinicalTrials identifier NCT01872936), a combinational therapy with telaprevir and ribavirin, two anti-viral drugs, was also performed for hepatitis C virus infection treatment [190].

Restoration of miR-205 by transiently overexpressing it, has been reported to play an anti-cancer role as demonstrated by inhibited cell proliferation, modulated EMT process, cell cycle arrest, induced apoptosis by the inhibition of cell survival and oncogenic pathways, tumor growth inhibition, and reversal of drug resistance (these actions were described above in detail). The EMT process is highly involved in cancer metastasis in addition to embryonic development and wound healing. A unique theoretical framework of miR-based coupled chimeric module identified that ZEB functions as a ternary switch for such phenotypic transitions [191].

**Figure 3:**
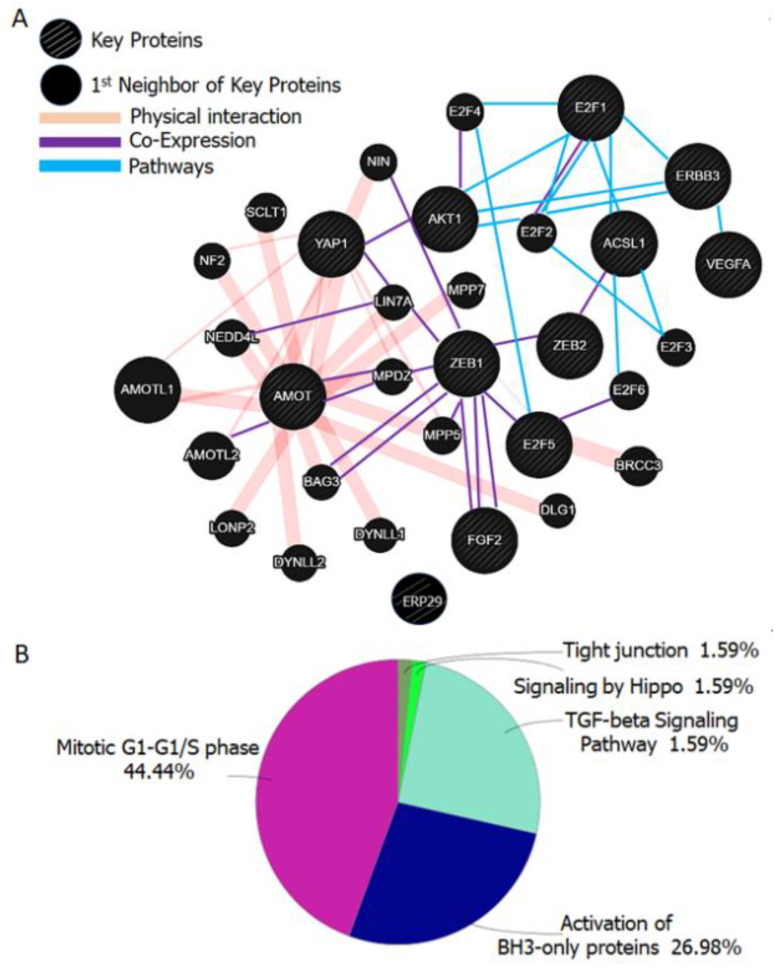
(**A**) Protein Interaction Network: Network generated by Cytoscape and GeneMANIA was used for the analysis of interaction network. Parameters that were used for networking: Max resultant genes were 20 and max resultant attributes were 10 and the weighing method was automatically selected from weighting methods. (**B**) Pathway enrichment analysis of key and neighboring proteins. Pathway enrichment analysis and Pie chart of 12 key and 20 neighboring proteins were generated by using ClueGO, a plug-in of Cytoscape (pV ≤ 0.05, with two-sided hypergeometric test and the pV correction is Bonferroni step down).

According to our intensive literature surveys, we found that ZEB1, ZEB2, AKT, E2F1, E2F5, VEGFA, YAP1, ACSL1, ERBB3, AMOT, ERp29, and FGF2 are the 12 key proteins which are well governed/regulated by miR-205 and are involved in various metabolic pathways. As per the anti-reductionism approach, it is assumed that proteins are well connected with each other in the form of a circuit and each protein in the group is accountable for regulating the various metabolic pathways [192]. By following all these concepts, we generated an miR-205 governed protein interactome (Figure 3A) by using the GeneMANIA web server [193], the number of total nodes were 22 and this interaction data was used for the pathway enrichment. Physical interaction, co-expression and pathways were selected as types of connections analysis (Figure 3B) by using ClueGO [194], a plug-in of Cytoscape [195]. Cytoscape and ClueGO are the software used for topological and pathway enrichment data analyses [196]. These previously published analyses helped us to identify the key interactome, enriched pathways, putative biomolecular targets, and interactors of our interest in a drug or molecule. Herein, data generated in this study indicated the 12 key proteins and their associated 20 proteins that are involved in various pathways such as Tight junction (AMOT, AMOTL1, AMOTL2, DLG1, MPDZ, MPP5, NEDD4L, NF2), Signaling by Hippo (AMOT, AMOTL1, AMOTL2, YAP1), TGF-beta Signaling Pathway (AKT1, E2F1, E2F2, E2F3, E2F4, E2F5, E2F6, FGF2, NEDD4L, YAP1, ZEB1, ZEB2), Activation of BH3-only proteins (AKT1, DYNLL1, DYNLL2, E2F1, E2F2, E2F3, E2F4, E2F5, FGF2, VEGFA) and Mitotic G1-G1/S phases (AKT1, E2F1, E2F2, E2F3, E2F4, E2F5, E2F6, FGF2, VEGFA, ZEB1). This type of enrichment study will open the new avenues for minute scrutinization of miR-205 associated proteins and their enriched pathways in cancer and other diseases.

Lai et al., [80] described systems biology-based investigation of cooperating the feasibility of various miRNAs as monotherapy or adjuvant therapy for various cancers. The results/discussion from this study can help in guiding the possible clinical trials development and potential miRNA therapies. This effort can also minimize off-targets in the treatment regimen. A significant therapeutic enhancement was noticed by utilizing a combination of miR-205 with miR-342 [197]. Another remarkable cooperative therapeutic benefit was also noticed due to the repression of E2F1 in aggressive melanoma and lung cancer cell lines. Previous systems biology-driven studies identified that E2F1 upregulation can minimize the effects of anti-PD1 (Programmed Cell Death 1) treatment. Thus, we can state that miR-205 may be an eligible candidate to reduce the E2F1 in cancer cells which may enhance the anti-PD1 therapy.

### 4.1. Additive Effects of miR-205 Therapy with Drugs

In most cases miRNAs are utilized in combination with chemotherapeutic drugs to achieve the improved therapeutic response. miR-205 has been documented as a chemosensitizer for many traditional drugs that are used in the clinics for cancer. The loss of miR-205 in cancer cells also leads to drug resistance. Reversal of its loss by restoration can normalize signaling pathways that are associated with carcinogenesis, tumorigenesis, and drug resistance. Hence, this section provides its influential roles in boosting anti-cancer potential of both genotoxic and cytostatic drugs. The above mentioned systems biology-based study identified several signature genes that are involved in chemoresistance. This study confirms that low expression level of miR-205 offers high expression of E2F1 and ERBB3. Additionally, low expression of miR-205 in tumors prepares the cells for resistance to conventional chemotherapies and cancer can even relapse after the therapy. As mentioned earlier that cooperativity of miRNAs can be synergistic if they can repress the common target(s). Such cooperative action lowers the total dose of miRNAs and chemotherapeutic agents.

Docetaxel is a standard first line chemotherapy for prostate and breast cancers. Puhr et al., [198] described that during the epithelial to mesenchymal transition, the expression level of E-cadherin was decreased and N-cadherin was increased in docetaxel resistant prostate cancer cells which further led to the loss of miR-205. Enhanced stemness and invasiveness in these cells were also associated with docetaxel resistance but when miR-205 was restored, EMT markers were modulated and docetaxel resistance was also rescued. In breast cancer, miR-205 showed synergistic effects with docetaxel treatment. Transient overexpression of miR-205 decreased the IC50 value of docetaxel in MDA-MB-231 and MCF-7 breast cancer cells from 6.35 to 1.95 and 5.74 to 1.26, respectively. Moreover, in combination with docetaxel, miR-205 synergistically inhibited the clonogenicity and tumor growth in these cells. Interestingly, higher concentration of docetaxel (8 µM) did not exhibit any improved/synergistic effects of miR-205 [199].

Cisplatin is a conventional chemotherapeutic drug in the clinics which is often associated with drug resistance [200]. Ectopic expression of miR-205 was found to hinder the autophagic events and sensitize castration resistant prostate cancer cells to cisplatin (CDDP) therapy as it induced the expression of LC3b-I and LC3b-II as well as it drastically decreased the clonogenic potential of these cells after cisplatin treatment [101]. Ultrasound Targeted Microbubble Destruction (UTMD) mediated delivery of miR-205 chemosensitized prostate cancer cells to cisplatin treatment. Overexpression of miR-205 further enhanced the growth inhibitory abilities of cisplatin as observed by a cell viability assay. Moreover, ectopic expression of miR-205 increased the apoptotic dead cells (PI and Annexin V positive) after cisplatin treatment from 5.42% (Cisplatin alone) to 26.88% (Cisplatin + UTMD mediated miR-205). Additionally, cisplatin’s ability to inhibit the cellular migration was further enhanced with miR-205 [201]. In cisplatin resistant glioma cells, overexpression of miR-205 reversed the drug resistance via targeting E2F1. miR-205 greatly enhanced the sensitivity of these cells to cisplatin therapy which resulted in improved inhibition of cell proliferation and cell cycle progression, as well as elevated expression of cleaved Caspase which is a clear sign of apoptosis induction [202].

Trastuzumab is an approved and widely used chemotherapy for breast cancer patients with HER2 and HER3 overexpression, however, due to the drug resistance more than half the people treated with this drug show cancer relapse [203,204]. In addition, a Patient Derived Xenograft model procured from trastuzumab resistant tumors confirmed the downregulated expression of miR-205 [205]. Therefore, to utilize miR-205 as a therapeutic tool for breast cancer, it was reported that overexpressed miR-205 inhibited the HER3 expression which as a result enhanced the trastuzumab sensitivity in breast cancer cells. Reversal of trastuzumab resistance by the restoration of miR-205 further increased the therapeutic effects of trastuzumab by reducing cell growth and blocking cell cycle progression [206].

A combination/cluster of miR-125a and miR-205 showed enhanced activity of trastuzumab and paclitaxel in HER2 overexpressing breast cancer cells. Overexpression of this cluster (miR-125a and miR-205) significantly promoted the cellular proliferation inhibition and cell cycle arrest, induced by trastuzumab. This combination showed a drastic increase in G1 phase cell cycle arrest (77.52%) and it also modulated the expression of cell cycle related proteins such as E2F1 (decreased), p27 and Cyclin D1 (both increased) after trastuzumab treatment. Moreover, this cluster of miR-125a and miR-205 enhanced the anti-cancer properties of paclitaxel by further promoting the apoptosis as confirmed through ELISA-apoptosis assay, and cleaved PARP and Caspases 3/8 [207].

Another report published on breast cancer revealed that miR-205 was significantly downregulated in two drug resistant breast cancer cell lines, MCF-7/A02 and CALDOX. In addition, its ectopic expression (stable cell lines with overexpressed miR-205) in these two cell lines significantly decreased the IC50 value of doxorubicin and taxol, indicating that multi-drug resistance was suppressed by miR-205 overexpression [43]. Collectively, these studies indicate that miR-205 acts as a chemosensitizer for many clinically used drugs, thus, exhibits the potential for easier and faster translation to the clinical settings.

### 4.2. Delivery of miRNAs Utilizing Nanotechnologies

miRNAs can function both as tumor suppressors and oncogenic miRNAs (oncomiRNAs) depending on their expression status in cancer. This duality of nature makes them more available to be utilized as a therapeutic tool for cancer management. For instance, suppressed miRNAs can be restored upon their successful delivery to the system and oncomiRNAs can be inhibited by anti-sense oligonucleotides or anti-cancer drugs. Despite having these mentioned approaches for the utilization of miRNAs as emerging therapeutics for cancer, their practical application is often associated with many limitations related to stability, non-specific targeting, and immune response stimulation. miRNAs are highly unstable and easily become degraded by the nucleases present in bodily fluids and blood [208]. Chemical modifications are often considered to increase the stability of miRNAs. However, simple chemical modifications may not be effective for in vivo translation. Therefore, efficient miRNA carriers are very much required for the successful development of miRNA therapies. Primarily, there are two types of miRNA delivery carriers, viral and non-viral delivery systems.

Viral vectors are a commonly applied method for the efficient transfer of the various genes, oligonucleotides, siRNAs, and miRNAs into various target cells or tissues/organs. Several viral vectors such as adenoviral, retroviral, and lentiviral vectors have been applied in preclinical and clinical evaluations. All these vectors are highly efficient in achieving higher delivery efficacy, however, their poor loading capacity, higher level of toxicity, and induction of immunogenicity limit their clinical translation. Thus, the development of non-viral vectors has received much attention for the successful and stable delivery of miRNAs. More insights of delivery aspects of various miRNAs delivery systems can be referred to in these reports [209,210].

One potential approach to safely deliver miRNAs and overcome these associated obstacles, is nanotechnology-based delivery [211]. Nanotherapy, in the beginning, was mainly aimed at delivering the anti-cancer drugs [212] but later on, many reports confirmed that nanoparticles can also successfully deliver nucleic acid molecules such as DNA, RNA and proteins/antibodies. miRNAs are negatively charged molecules with large size which hinders their ability to cross cell membranes but literature has evidenced that positively charged polymers such as poly(ethyleneimine) and/or cationic based liposomes or polymers, can remarkably control this barrier of miRNAs [213]. Among many non-viral vectors, lipid-based delivery systems have efficiently complexed or encapsulated miRNAs inside the lipoplex/liposome membrane-like surface. This category-based delivery system is highly popular and there are a few commercially available vectors existing. These include Lipofectamine^®^, SiPORT™, DharmaFECT^®^ and SilentFect™ [209,210]. However, liposomal carriers have some limitations such as their short half-lives and extensive binding with serum proteins which minimize the effective delivery of miRNAs to the intended tissue/organ. A newer way of delivery, utilizing the polymer-based carriers (PLGA, PEI, or dendritic polymers), can be useful for the efficient delivery of miRNAs with minimal toxicity in cells. A cell-penetrating peptide can also be a suitable vehicle for miRNA delivery purposes. Extracellular vesicles (exosomes or endosomal vesicles) have also been in the use for the delivery application of siRNAs and miRNAs [214]. However, limited work has been reported using these non-viral vectors for miR-205 delivery.

**Figure 4:**
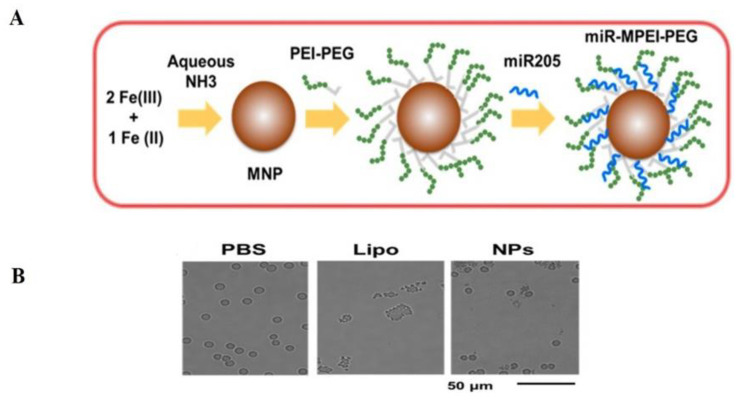
(**A**) Preparation of miR-205 loaded MNP nanoformulation. (**B**) Hemolysis images of miR-205 MNP nanoformulation. Microscopic images showing hemolytic characteristic of human red blood cells. This figure was adopted from our previous publication [215].

Owing to the tumor suppressive role of miR-205 in prostate cancer described in multiple studies, our laboratory has utilized a nanotherapy based approach to improve the delivery and therapeutic efficacy of miR-205 in prostate cancer [215]. We have already established a preparation technique/method in our laboratory for the successful generation of MNP nanoformulations [216]. Following this method, a PEI–PEG conjugated MNP (magnetic nanoparticles) formulation was prepared (Figure 4A) with a particle size of about 100 nm which made it ideal for tumor delivery via the Enhanced Permeation and Retention (EPR) effect. The prepared miR-205 loaded MNP nanoformulation was safe to use in the cellular system as evidenced by hemolysis assay where red blood cells were captured in a healthy state after the treatment with this formulation compared to lipofectamine-delivered miRNA (Figure 4B).

This formulation exhibited superior cellular internalization via endocytosis, escaping the endosomal and lysosomal degradation in C4-2 and PC-3, two prostate cancer cell lines. Moreover, we used this novel MNP miR-205 formulation in combination with docetaxel which is an FDA approved therapy for prostate cancer. Restoration of miR-205 successfully reversed the drug resistance and sensitized the prostate cancer cells towards docetaxel treatment. Furthermore, in this combination therapy, miR-205 MNP nanoformulation demonstrated an additive effect on anti-prostate cancer properties of docetaxel as observed by significantly reduced cell proliferation, migration, invasion, and induced apoptosis [215].

Another nanoformulation of miR-205 based on gold nanoparticles was reported to reintroduce miR-205 in prostate cancer cells. Restoration of miR-205 decreased the levels of Protein Kinase C Epsilon (PKCε) and regulated cellular processes [217]. In 2014, Mittal et al., [173] prepared a cationic co-polymeric formulation (micelle) to co-deliver miR-205 and gemcitabine in pancreatic cancer cells/tumors. miR-205 was evident to be significantly suppressed in gemcitabine resistant MIA PaCa-2 pancreatic cancer cell line. This micelle formulation showed a greater stability to miR-205 and had particles size range between 62 nm and 122 nm. Upon the treatment with gemcitabine conjugated co-polymeric miR-205 formulation, gemcitabine resistant pancreatic cancer cells became more sensitive to the drug treatment and resulted in the reduced cell growth. Moreover, expression levels of E-cadherin were upregulated and ZEB1 were downregulated as miR-205 was restored in these cells which in result made these cells less motile and invasive. Furthermore, as drug resistance was reversed in these cells by miR-205, in vivo findings were consistent with the cellular data, tumor growth and weight were significantly reduced after gemcitabine–miR-205 complexed formulation treatment [173]. Therefore, nanoformulation based delivery of miR-205 promises an improved and targeted therapeutic outcome for cancer management.

## 5. Conclusions

**Figure 5:**
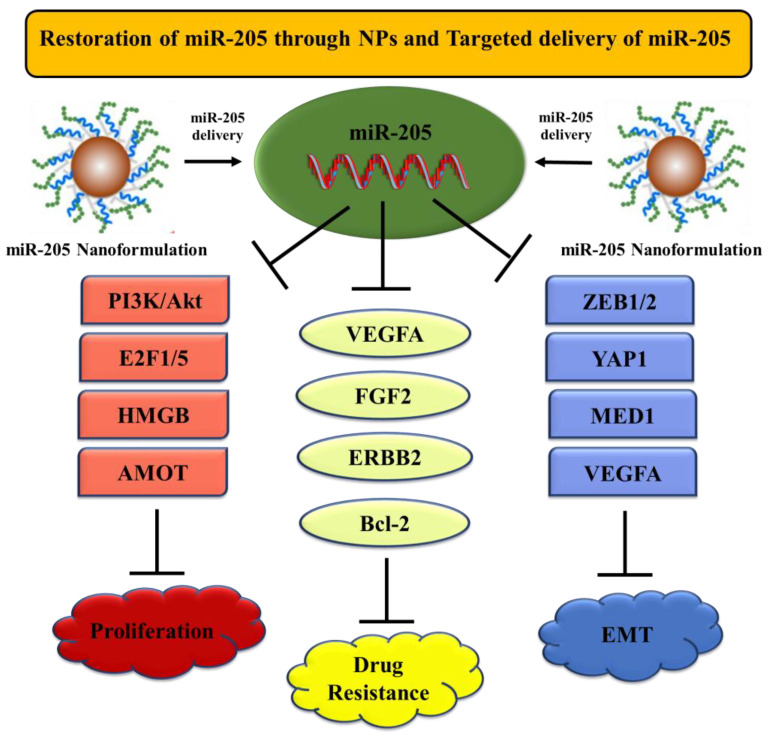
Schematic diagram showing restoration of miR-205 through nanoparticles. Delivery of miR-205 with nanoformulations can lead to an enhanced tumor suppressive role of miR-205 in cancer via targeting several signaling pathways/genes.

miR-205 is a dual nature (can act as both, oncogene and tumor suppressor) miRNA whose role in a specific cancer depends on its expression status. In this review, we explained the tumor suppressive role of miR-205 in several cancers. miR-205 is notably involved in the pathogenesis of many cancers and facilitates its tumor suppressive action by regulating numerous genes/pathways. Loss of miR-205 is not only associated with tumor progression but also correlates with poor overall survival of cancer patients. miR-205 has an established regulatory role in cancer migration and invasion via targeting EMT markers such as E-cadherin and N-cadherin. Moreover, ZEB1 and 2 were identified as direct targets for miR-205. miR-205 modulates the expression of AKT and VEGFA signaling pathways, and as a result, regulates cellular proliferation, cell cycle, and apoptosis in a variety of cancers. Collectively, miR-205 is a multi-functional miRNA that targets different pathways/genes in cancer, therefore, acts as a potent diagnostic and prognostic biomarker. Herein, utilizing a nanotherapy based approach, miR-205 can successfully be delivered to the cells and can be utilized as a therapeutic tool to achieve improved overall cancer management (Figure 5).

## Figures and Tables

**Table 1:** Expression status, targeted genes, and resulted functions of miR-205 in different cancers in comparison with the corresponding normal tissue(s).

Cancer	Expression Status	Target	Function	References
Breast	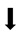	Erb-B2 Receptor Tyrosine Kinase 3 (*ERBB3*), Vascular Endothelial Growth Factor A (*VEGFA*), Fibroblast Growth Factor 2 (*FGF2*), Phosphatase and Tensin Homolog (*PTEN*), E2F Transcription Factor 1 (*E2F1*), *E2F5*, Zinc Finger E-Box Binding Homeobox 1 (*ZEB1*), and *ZEB2*	Proliferation, Migration, and Invasion	[28,40,41,42,43]
Cervical	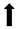	Cellular Communication Network Factor 1 (*CCN1*) and *CCN2*	Proliferation and Migration	[27]
Ovarian	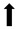	Transcription Factor 21 (*TCF21*), Matrix Metallopeptidase 2 (*MMP2*), *MMP10*, *PTEN*, and SMAD Family Member 4 (*SMAD4*)	Invasion and Proliferation	[44,45,46]
Liver	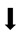	Cadherin 1 (*CDH1*), *ZEB1*, *ZEB2* Acyl-CoA Synthetase Long Chain Family Member 1 (*ACSL1*), and *ACSL4*	Migration, Invasion, and Lipid Metabolism	[42,47,48]
Glioblastoma	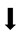	Yes1 Associated Transcriptional Regulator (*YAP1*) and *VEGFA*	Proliferation, Migration, Invasion, Cell Cycle, and Apoptosis	[49,50]
Endometrial	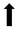	*PTEN,* Estrogen Related Receptor Gamma (*ESRRG*), AKT Serine/Threonine Kinase (*AKT*), and Mechanistic Target Of Rapamycin Kinase (*MTOR*)	Poor Patients Survival, Growth, Cell Cycle, Apoptosis, and Autophagy	[51,52,53]
Pancreatic	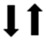	Runt-Related Transcription Factor 2 (*RUNX2*), Tubulin Beta 3 Class III (*TUBB3*), Ribonucleotide Reductase Catalytic Subunit M1 (*RRM1*), and *ZEB1*	Proliferation, Migration, Invasion, and Drug Resistance	[54,55]
Prostate	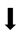	Androgen Receptor (*AR*), Mitogen-Activated Protein Kinase 1 (*MAPK1*), *CDH1*, Interleukin 24 (*IL24*) and *IL32*	Proliferation, Clonogenicity, Migration, Invasion, and Adhesion	[28,56,57,58]
Lung	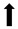	*SMAD4*, *PTEN*, PH Domain and Leucine Rich Repeat Protein Phosphatase 2 (*PHLPP2*), and *AKT*	Proliferation, Cell Cycle, Tumor Growth, Metastasis, and Chemoresistance	[59,60,61]
Skin	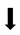	*E2F1*, *AKT*, *ZEB1*, and *ZEB2*	Proliferation and EMT	[62,63]
Renal	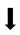	SRC Proto-Oncogene, Non-Receptor Tyrosine Kinase (*SRC*), Mitogen-Activated Protein Kinase 3 (*MAPK3*), (*MAPK1*), Protein Tyrosine Kinase 2 (*PTK2*), Signal Transducer and Activator of Transcription 3 (*STAT3*), cluster of differentiation 1 (*CD1*), MYC Proto-Oncogene, BHLH Transcription Factor (*MYC*), *VEGFA*, and Egl-9 Family Hypoxia Inducible Factor 2 (*EGLN2*)	Reactive Oxygen Species, Cell Cycle, Proliferation, Migration, and Invasion	[64,65]
Colorectal	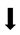	cAMP Responsive Element Binding Protein (*CREBP*)	Proliferation, Migration, and Invasion	[66]
